# Supplementary material for: Antioxidant and Anticancer Activity of Vitis vinifera Extracts in Breast Cell Lines
Source: Life (Basel). 2024 Feb 6;14(2):228. doi: 10.3390/life14020228 (PMC10890198; doi:10.3390/life14020228)
Supplement: Supplementary file 1 [file life-14-00228-s001.zip › Supplementary Data.pptx]

## Slide 1
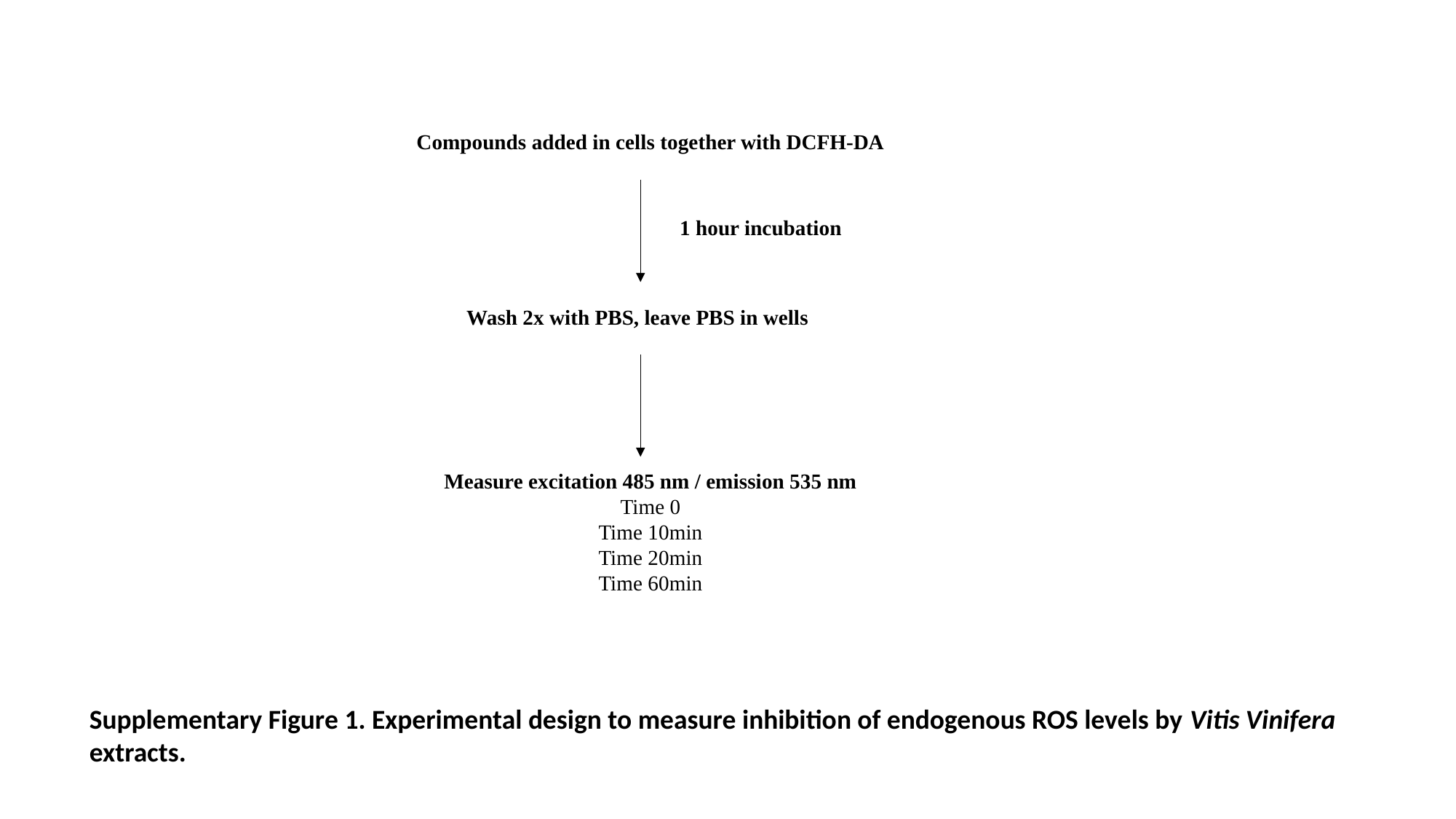

Compounds added in cells together with DCFH-DA
1 hour incubation
Wash 2x with PBS, leave PBS in wells
Measure excitation 485 nm / emission 535 nm
Time 0
Time 10min
Time 20min
Time 60min
Supplementary Figure 1. Experimental design to measure inhibition of endogenous ROS levels by Vitis Vinifera extracts.

## Slide 2
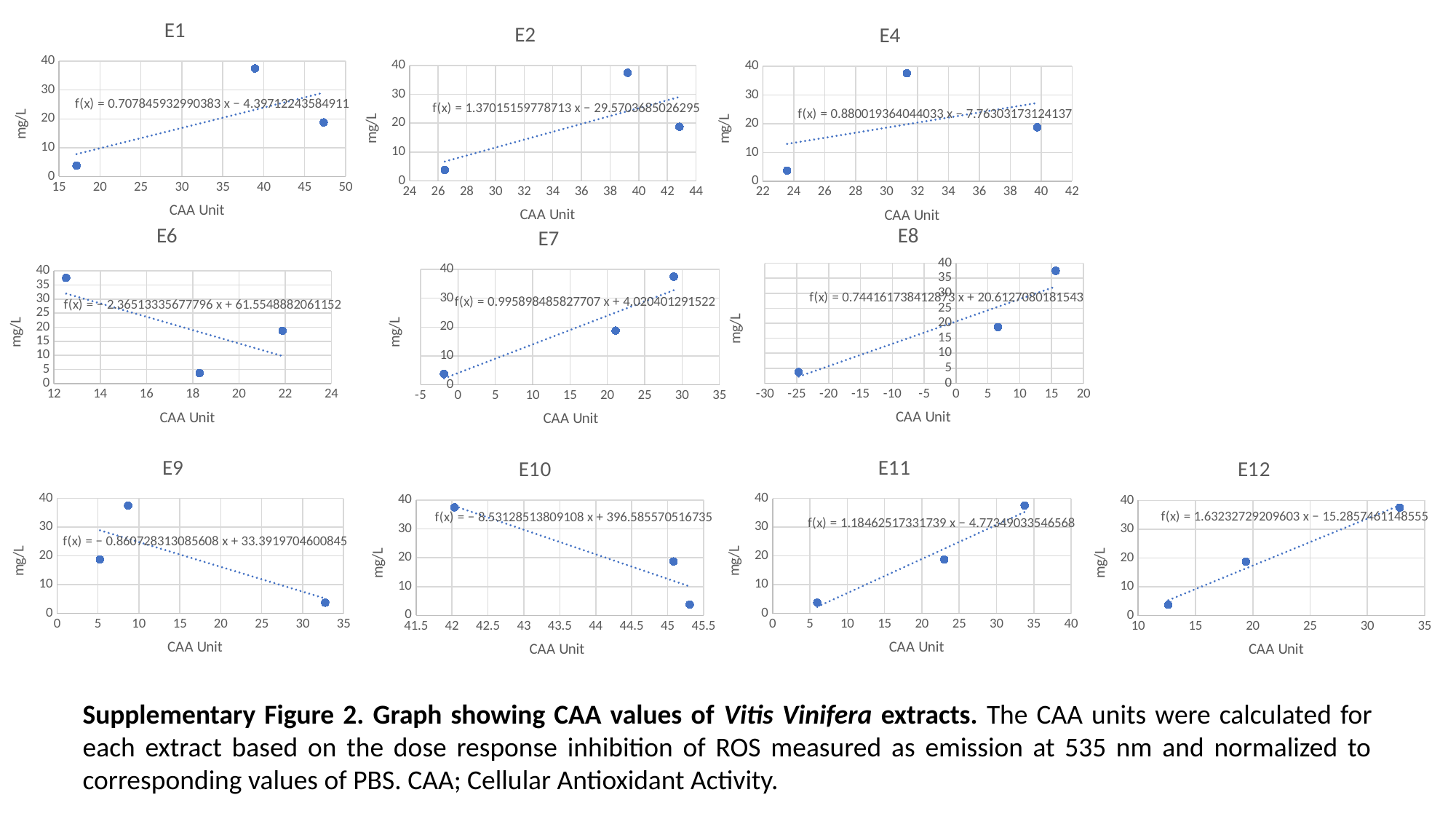

### Chart: E1
| Category | |
|---|---|
### Chart: E2
| Category | |
|---|---|
### Chart: E4
| Category | |
|---|---|
### Chart: E6
| Category | |
|---|---|
### Chart: E8
| Category | |
|---|---|
### Chart: E7
| Category | |
|---|---|
### Chart: E9
| Category | |
|---|---|
### Chart: E11
| Category | |
|---|---|
### Chart: E10
| Category | |
|---|---|
### Chart: E12
| Category | |
|---|---|Supplementary Figure 2. Graph showing CAA values of Vitis Vinifera extracts. The CAA units were calculated for each extract based on the dose response inhibition of ROS measured as emission at 535 nm and normalized to corresponding values of PBS. CAA; Cellular Antioxidant Activity.
